# Association between IRF6 and 8q24 polymorphisms and nonsyndromic cleft lip with or without cleft palate: Systematic review and meta‐analysis

**DOI:** 10.1002/bdra.23540

**Published:** 2016-08-11

**Authors:** Kachin Wattanawong, Sasivimol Rattanasiri, Mark McEvoy, John Attia, Ammarin Thakkinstian

**Affiliations:** ^1^ Section for Clinical Epidemiology and Biostatistics, Faculty of Medicine, Ramathibodi Hospital, Mahidol UniversityBangkokThailand; ^2^ Department of SurgeryFaculty of Medicine, Ramathibodi Hospital, Mahidol UniversityBangkokThailand; ^3^ Centre for Clincial Epidemiology and Biostatistics, School of Medicine and Public Health, Faculty of Health and Medicine, University of Newcastle, and Hunter Medical Research InstituteNSWAustralia

**Keywords:** IRF6, 8q24, nonsyndromic cleft lip with or without cleft palate, meta‐analysis, polymorphism

## Abstract

**Background:**

We conducted a systematic review and meta‐analysis of interferon regulatory factor 6 and 8q24 polymorphisms with nonsyndromic cleft lip with/without cleft palate (NSCL/P).

**Methods:**

Data extraction was independently performed by two reviewers. Genotypic effects of four polymorphisms from 31 studies were pooled separately by ethnicity using a mixed‐effect logit model with accounting for heterogeneity.

**Results:**

For rs2235371, AA and GA carried, respectively, 51% (95% confidence interval [CI], 37%–61%) and 42% (95% CI, 32%–50%) lower risks of NSCL/P than GG genotypes in Asians, but these genotypes were not significant in Caucasians. For rs2013162, only AA was significant, that is, carried 0.65 (95% CI, 0.52–0.82) times lower odds than CC in Caucasians but not for Asians. For rs642961, AA and GA genotypes, respectively, carried 2.47 (95% CI, 1.41–4.35) and 1.40 (95% CI, 1.12–1.75) times higher odds in Asian, and 2.03 (95% CI, 1.52–2.71) and 1.58 (95% CI, 1.37–1.82) times higher odds in Caucasians compare with GG genotypes. For rs987525, AA and CA genotypes carried 2.27 (95% CI, 1.43–3.60) and 1.34 (95% CI, 1.02–1.77) times higher odds in Asian, and 5.25 (95% CI, 3.98–6.91) and 2.13 (95% CI–1.82, 2.49) times higher odds in Caucasians, and 1.42 (95% CI, 1.10–1.82) and 1.28 (95% CI, 1.09–1.50) times higher odds in mixed ethnicities compared with CC genotypes. These variant effects remained significant based on applying Bonferroni corrected‐thresholds, except in the mixed ethnicity.

**Conclusion:**

We show robust variant effects in NSCL/P. Considering them with other genes and risk factors might be useful to improve prediction of NSCL/P occurrence. Birth Defects Research (Part A) 106:773–788, 2016. © 2016 The Authors Birth Defects Research Part A: Clinical and Molecular Teratology Published by Wiley Periodicals, Inc.

## Introduction

Nonsyndromic cleft lip with or without cleft palate (NSCL/P) is one of the commonest birth malformations and varies by geographic origin and ethnicity (Bender, 2000). The incidence is approximately 1 in 500 live births in Asia and America, 1 in 1000 in Europe, and 1 in 2500 in Africa. Cleft lip without cleft palate is more frequent in males than females, with a ratio of 2:1, and frequently occurs on the left rather than right side (Mossey et al., 2009).

Patients with NSCL/P suffer from functional and cosmetic problems, and NSCL/P can affect speech and communication, which results in delayed development, low self‐confidence, and poor quality of life for both patient and family members. Although reconstruction with maxillo‐facial surgery is possible, severe cases sometimes require multiple corrective surgeries (e.g., cheiloplasy, palatoplasty, rhinoplasty, orthognathicsurgery), dental work, and speech rehabilitation. Thus, this condition places a significant burden on family and medical services (Strauss, 1999; Murray, 2002).

There have been attempts to identify disease genes associated with NSCL/P as identification of disease genes may shed light on the etiology of the condition and facilitate efforts at prevention of disease. Mutations of interferon regulatory factor 6 (*IRF6*), located on chromosome ***1q32.2*** (OMIM # 607199), is associated with the autosomal‐dominant Van der Woude syndrome, the most common Mendelian syndrome that has the cardinal signs of cleft lip with or without cleft palate (CL/P) and/or cleft palate only (CPO) with dental anomalies and pitted lips (Kondo et al., 2002; Rizos and Spyropoulos, 2004). Polymorphisms in *IRF6* were later identified by Zucchero et al. (2004) and replicated by individual studies (Zucchero et al., 2004; Rahimov et al., 2008; Birnbaum et al., 2009b) and genome‐wide association studies (Birnbaum et al., 2009b; Grant et al., 2009; Beaty et al., 2010; Ludwig et al., 2012), as associated with NSCL/P. Animal studies also indicate that IRF6 is involved in proliferation‐differentiation of the keratinocyte (Richardson et al., 2009), and hyper‐proliferation of the epidermis may result in failure of terminal differentiation and multiple epithelial adhesion leading to CL/P (Ingraham et al., 2006; Richardson et al., 2006).

A genome‐wide association study of German subjects of Central European origin identified 146 polymorphisms in the 8q24 region, of which rs987525 (OMIM # 612858) was the most significantly associated with NSCL/P and was the major susceptibility locus in the general population (Birnbaum et al., 2009b). Grant et al. (2009) also conducted a case–control genome‐wide association studies (GWAS) in the United States and confirmed the associations of this region. In addition, Beaty et al. (2010) had replicated the GWAS in Asian subjects and had similar results, that is rs987525 was the most significant polymorphism. However, the biological mechanism of this SNP remains unclear.

Several independent studies replicated the effects of the *IRF6* gene (at rs2235371 and rs642961) and the 8q24 region (at rs987525) on NSCL/P, and their effects were later pooled by a meta‐analysis (Wang et al., 2012). However, this meta‐analysis pooled only allele effects rather than genotypic effects, which assumes an additive mode of action. In addition, further studies have been published since then. We, therefore, performed an updated meta‐analysis on the same polymorphisms with additional *IRF6* polymorphisms at rs2013162 aiming to assess both variant effects and possible genetic models.

## Materials and Methods

### Search Strategy

This review was conducted following the HuGE Review guideline (Little and Higgins, 2006; Gwinn et al., 2014). Studies were identified by two reviewers (K.W. and S.R.) using Medline (by means of PubMed) and Scopus (Sci Verse Scopus; Elsevier B.V.) databases up to February 15, 2016. Search terms used were as follows: (irf6 or irf‐6 or “interferon regulatory factor 6” or “interferon regulation factor‐6” or 8q24) and (polymorphism or gene or allele or SNP) and (NSCL/P or “cleft lip” or CL or “cleft palate” or CP or CPO or “nonsyndromic cleft”). Details of the search strategies are described in Supplementary Table 1, which is available online.

### Inclusion and Exclusion Criteria

Two reviewers (K.W. and S.R.) independently selected studies by looking through all titles and abstracts of the identified studies. Any human population‐based (not familial‐based) association study published in English was included if it met the following criteria: the outcome of interest was NSCL/P, the studied polymorphisms were in *IRF6* or 8q24, and sufficient data were reported, that is, allele/genotype frequency between case and control; or odds ratio (OR) of allele/genotype effects on outcome along with its 95% confidence interval (CI). The studies with insufficient information were excluded if authors did not provide data after two contacts. Where there were multiple publications with the same or overlapping subjects (which could be detected by incorporating information on authorship lists, setting, study period, genotyping method, and subjects) the most complete and/or recent results were used. The reference lists of the selected articles were also reviewed to identify additional relevant publications.

### Data Extraction

Summary data for *IRF6* and 8q24 were extracted independently by two reviewers (K.W. and S.R.) using a standardized data extraction form. Data on co‐variables, for example, mean age, percentage of males, ethnicity, and family history were also extracted. The main data (generic data and risk of bias assessment) were computerized separately by reviewer. Then, the two data were compared and validated to identify disagreement between. Meetings between the two reviewers (K.W. and S.R.) and the third party (A.T.) were then setup to discuss and solve disagreement.

### Risk of Bias Assessment

The quality of studies was independently assessed by 2 reviewers (K.W. and S.R.) using a risk of bias assessment for genetic association studies, described in detail previously (Thakkinstian et al., 2005b, 2011). Briefly, the assessment considered five domains: selection bias, information bias, population stratification, selective reports, and assessment of Hardy‐Weinberg equilibrium (HWE). Each item was classified as yes, no, or unclear, corresponding to low risk of bias, high risk of bias, or unclear/insufficient information. Disagreement was solved by discussion with senior author (A.T.).

### Outcome and Studied Gene of Interests

Our review focused on NSCL/P as the outcomes of interest, which diagnosed according to original studied. The studied genes were three polymorphisms within the *IRF6* gene (rs2235371 G>A, rs642961 G>A, and rs2013162 C>A), and one located within 8q24 (rs987525 C>A).

### Statistical Analysis

Data analysis was performed following a standard method of meta‐analysis for genetic association studies (Thakkinstian et al., 2005a, 2012). HWE was checked in control groups for case‐control studies using the Fisher's exact test. Data were then pooled based on studies that complied with HWE and if there were at least three studies for each studied polymorphism. A prevalence of minor allele frequency (MAF) for each polymorphism was estimated for each study, and then pooled across studies separately by ethnicity (Thakkinstian et al., 2005b).

Variant effects on NSCL/P were assessed using a per‐allele and per‐genotype approach. For the per‐allele approach, an OR of a versus A, if a and A were, respectively, minor and major alleles, along with its variance were estimated. Heterogeneity was then assessed using Cochrane's Q test and *I^2^* statistic. The ORs were then pooled using a random‐effect model by Der Simonian & Laird method if heterogeneity was present (*p* value < 0.10 or *I^2^* ≥ 25%); otherwise a fixed‐effect model with inverse‐variance method was applied.

For the per‐genotype approach, OR_1_ (aa vs. AA) and OR_2_ (Aa vs. AA) were estimated for each polymorphism across included studies. Heterogeneity was then assessed using the same methods described above. If any of the two ORs was heterogeneous, data were then pooled using a mixed‐effect hierarchical logit model (Thakkinstian et al., 2012). In this model, the genotypes were considered as fixed effects, whereas the study was considered as a random effect. A likelihood ratio test was used to assess whether an overall variant effect was significant. The pooled ORs along with 95% CI were then estimated by exponential coefficients of the mixed‐effect logit model. If the overall variant effect was present, the mode of genetic effect was captured by the parameter lambda (λ = logOR_2_ /logOR_1_), which was then estimated using the model‐free Bayesian approach (Minelli et al., 2005). The estimated λ (range: 0 to 1) would suggests a recessive, dominant, and additive effect if λ closes to 0, 1, and 0.5, respectively.

A sensitivity analysis was performed by including studies not observing HWE in the main pooling. Publication bias was assessed using the Egger's test and funnel plot (Egger et al., 1997). In addition, contour enhanced‐funnel plots were generated if there was any evidence of asymmetry suggested by funnel plot or Egger's test (Peters et al., 2008).

Analyses were performed using STATA version 14 (StataCorp, 2013; van Rooij et al., 2004) and WinBugs 1.4.2 (Spiegelhalter et al., 2007) with normal vague prior distributions for estimation of lambda and ORs. The model was run with 1000 iteration burn in, following by 10,000 iterations for estimation of parameters. A *p*‐value less than 0.05 was considered statistically significant, except for tests of heterogeneity where a level of 0.10 was used. In addition, Bonferroni correction was applied to adjust for 27 multiple tests in total (see Supplementary Table 2), which resulted in a Bonferroni corrected threshold of 1.85 × 10^‐3^.

## Results

### Identifying Studies

**Figure 1:**
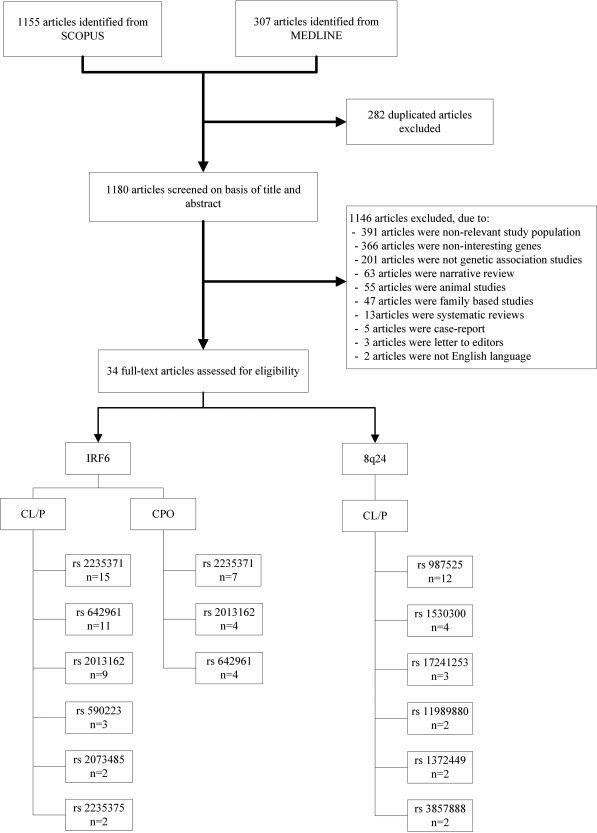
Flow chart for searching.

A total of 307 and 1155 studies were, respectively, located from Medline by means of PubMed and Scopus (Fig. 1). After 282 duplicates were removed, 1180 studies were screened on titles or abstracts, and 34 studies were eligible. Reasons for ineligibility are provided in Figure 1, but were mainly nonrelevant genes or populations, and nongenetic association studies. For studies meeting inclusion criteria, the study by Letra et al. (2012) had insufficient data but the author provided additional data after our request. Studied polymorphisms and number of studies for each polymorphism are described in Figure 1. Among them, three polymorphisms (i.e., rs2235371, rs2013162, rs642961) for *IRF6*, and one polymorphism (i.e., rs987525) for 8q24 had sufficient numbers of studies; therefore, further pooling was focused on only these polymorphisms from 31 studies.

The characteristics of these 31 studies (Srichomthong et al., 2005; Rahimov et al., 2008; Jugessur et al., 2008; Ali et al., 2009; Birnbaum et al., 2009a, 2009b; Grant et al., 2009; Huang et al., 2009; Nikopensius et al., 2009; Tang et al., 2009; Carter et al., 2010; Mostowska et al., 2010; Pan et al., 2010; Paranaiba et al., 2010; Rojas‐Martinez et al., 2010; Shi et al., 2011; Weatherley‐White et al., 2011; Brito et al., 2012a, 2012b; Letra et al., 2012; Xu et al., 2012; Velazquez‐Aragon et al., 2012; Hikida et al., 2012; Bagordakis et al., 2013; Song et al., 2013; Lu et al., 2013; Aldhorae et al., 2014; Krasone et al., 2014; do Rego Borges et al., 2015; Kerameddin et al., 2015; Mijiti et al., 2015) are given in Table 1. All studies were case–control designs. Thirteen, 11, and 7 studies were conducted in Asian, Caucasian, and mixed populations. Mean age ranged from approximately 2.6 to 17.3 years, and percent male ranged from 46% to 74%. Fifteen, 9, 11, and 12 studies reported data on rs2235371, rs2013162, rs642961 for *IRF6*, and rs987525 for 8q24, with the outcome NSCL/P, respectively.

**Table 1:** Characteristics of Included Studies

Author	Year	Country	Ethnicity	Study design	SNP	Locus	Percentage male	Percentage CLO
Srichomthong C.	2005	Thai	Asian	Case‐control	rs2235371	*IRF6*	0.58	0.38
Jugessur A.	2008	Norway	Caucasian	Case‐control	rs2235371	*IRF6*		
					rs2013162	*IRF6*		
Rahimov F.	2008	Norway, Denmark	Caucasian	Case‐control	rs642961	*IRF6*		0.4
Ali A.	2009	India	Asian	Case‐control	rs2235371	*IRF6*	0.7	
Birnbaum S.	2009	Central Europe	Caucasian	Case‐control	rs2235371	*IRF6*		
					rs2013162	*IRF6*		
					rs642961	*IRF6*		
Birnbaum S.	2009	Central Europe	Caucasian	Case‐control	rs987525	8q24		0.22
Grant S.	2009	USA	Caucasian	Case‐control	rs987525	8q24	0.69	
Huang Y.	2009	China	Asian	Case‐control	rs2235371	*IRF6*		0.15
					rs2013162	*IRF6*		
Nikopensius T.	2009	Estonia, Lithonia	Caucasian	Case‐control	rs987525	8q24		
Tang W.	2009	China	Asian	Case‐control	rs2235371	*IRF6*	0.74	
Carter TC.	2010	Ireland	Caucasian	Case‐control	rs2235371	*IRF6*		
					rs2013162	*IRF6*		
Mostowska A.	2010	Poland	Caucasian	Case‐control	rs642961	*IRF6*	0.51	0.11
					rs987525	8q24		
Pan Y.	2010	China	Asian	Case‐control	rs2235371	*IRF6*	0.64	0.4
					rs642961	*IRF6*		
Paranaiba L.	2010	Brazil	Caucasian	Case‐control	rs2235371	*IRF6*		
					rs642961	*IRF6*		
Rojas‐Martinez A.	2010	Mexico	Mixed	Case‐control	rs987525	8q24		

Author	Year	Country	Ethnicity	Study design	SNP	Locus	Mean age	Percentage male	Percentage CLO
Shi J.	2011	China	Asian	Case‐control	rs2235371	*IRF6*	1 to 28	0.46	0.29
					rs642961	*IRF6*			
Weatherley‐White R.	2011	Kenya	Mixed	Case‐control	rs2013162	*IRF6*			
					rs987525	8q24			
Brito L.	2012	Brazil	Mixed	Case‐control	rs642961	*IRF6*			0.19
Brito L.	2012	Brazil	Mixed	Case‐control	rs987525	8q24			
Hikida M.	2012	Japan	Asian	Case‐control	rs987525	8q24			
Letra A.	2012	Brazil	Caucasian	Case‐control	rs2235371	*IRF6*	17.32	0.6	
					rs2013162	*IRF6*			
Velazquez‐Aragon J.	2012	Mexico	Mixed	Case‐control	rs2235371	*IRF6*			
					rs987525	8q24			
Xu M.	2012	China	Asian	Case‐control	rs987525	8q24	7.95	0.68	0.2
Bagordakis E.	2013	Brazil	Mixed	Case‐control	rs2013162	*IRF6*			0.35
Lu Y.	2013	China	Asian	Case‐control	rs2235371	*IRF6*			
					rs2013162	*IRF6*			
Song T.	2013	China	Asian	Case‐control	rs2235371	*IRF6*			
Aldhorae KA.	2014	Arab	Asian	Case‐control	rs642961	*IRF6*			
					rs987525	8q24			
Krasone K.	2014	Latvia	Caucasian	Case‐control	rs642961	*IRF6*			
Do Rego Borges A.	2015	Brazil	Mixed	Case‐control	rs642961	*IRF6*			
					rs987525	8q24			
Kerameddin S.	2015	Iran	Asian	Case‐control	rs642961	*IRF6*			
Mijiti A.	2015	China	Asian	Case‐control	rs2235371	*IRF6*			
					rs2013162	*IRF6*			

### Risk of Bias Assessment

The results of bias assessment are presented in Supplementary Table 3. All studies had low risks of bias related to ascertainment of NSCL/P and population stratification. Twenty‐nine of 31 studies assessed HWE. Most studies (97%) had low risk of selective reporting of outcomes. Twenty‐seven (87%) and 24 (77%) studies described control ascertainment and quality control for genotyping, respectively.

### rs2235371

Fifteen studies (i.e., 9 Asians, 5 Caucasians, and 1 mixed ethnicity) assessed effect of rs2235371 polymorphism on NSCL/P. Allele frequencies in the NSCL/P and control groups are described, and genotype frequencies in the control groups complied with HWE for all studies (Supplementary Table 4). The MAF was pooled by ethnicity. This suggested the pooled prevalence of MAF A in Asians was 0.34 (95% CI, 0.28–0.40) in the control group and 0.26 (95% CI, 0.19–0.32) in NSCL/P groups, yielding a pooled OR of 0.66 (95% CI, 0.58–0.75, *p* = 1.27 × 10^‐9^), which was still significant based on Bonferroni corrected‐threshold. The MAF A in Caucasians was very rare and much lower than in Asians, that is, 0.03 (95% CI, 0.02–0.04) and 0.02 (95% CI, 0.01–0.04) in control and NSCL/P groups, respectively. This yielded a pooled OR of 0.69 (95%CI, 0.43–1.12; *p* = 0.131), but this was not statistically significant.

**Figure 2:**
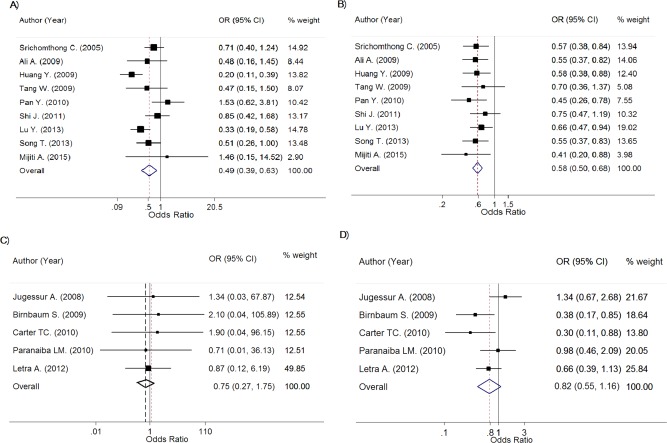
Forest plots for rs2235371 of IRF6.

The genotype frequencies between NSCL/P (*n* = 3513) and control (*n* = 4756) groups along with ORs for all studies are described in Table 2. Because MAF was different between Asians and Caucasians, pooling genotype effect was, therefore, performed separately by ethnicity. Degrees of heterogeneity *I^2^* for OR_1_ (AA vs. GG) and OR_2_ (GA vs. GG) were, respectively, 60.0% (Chi‐square = 19.98; d.f. = 8; *p* = 0.010) and 0% (Chi‐square = 3.70; d.f. = 8, and *p* = 0.883) for pooling 9 Asian studies (n = 1677 vs. 1717). The corresponding pooled ORs were 0.49 (95% CI, 0.39–0.63; *p* = 7.73 × 10^‐9^) and 0.58 (95% CI, 0.50–0.68; *p* = 1.50 × 10^‐12^), see Figure 2A,B. These genotype effects were still significant after applying Bonferroni corrected‐threshold, indicating that carrying AA and GA is associated with approximately 51% and 42% lower odds of having NSCL/P than carrying GG genotype. The estimated lambda was 0.71 (95% CI, 0.38–0.98), indicating that a mode of variant effect could be from additive to dominant effect.

**Table 2:** Genotype Frequencies and Genotype Effects for IRF‐6 at SNPs 2235371 Between NSCL/P and Control Groups

		CL/P				Control							
	Year	No. of subjects	Genotype			No. of subjects	Genotype			AA/GG		GA/GG	
Author			GG	GA	AA		GG	GA	AA	OR_1_	95% CI	OR_2_	95% CI
Asian													
Srichomthong C.	2005	192	93	72	27	278	100	137	41	0.71	0.40, 1.24	0.57	0.38, 0.84
Ali A.	2009	323	252	65	6	214	141	66	7	0.48	0.16, 1.46	0.55	0.37, 0.82
Huang Y.	2009	257	133	106	18	174	57	79	38	0.20	0.11, 0.39	0.58	0.38, 0.88
Tang W.	2009	66	30	31	5	96	34	50	12	0.47	0.15, 1.50	0.70	0.36, 1.37
Pan Y.	2010	127	69	40	18	115	47	60	8	1.53	0.62, 3.81	0.45	0.26, 0.78
Shi J.	2011	173	82	69	22	154	63	71	20	0.85	0.42, 1.68	0.75	0.47, 1.19
Lu Y.	2013	236	122	96	18	400	152	180	68	0.33	0.19, 0.58	0.66	0.47, 0.94
Song T.	2013	203	114	72	17	226	93	106	27	0.51	0.26, 1.00	0.55	0.37, 0.83
Mijiti A.	2015	100	80	17	3	60	39	20	1	1.46	0.15, 14.52	0.41	0.20, 0.88
Pooled										0.49	0.39, 0.63	0.58	0.50, 0.68
Caucasian													
Jugessur A.	2008	314	297	17	0	416	399	17	0	1.34	0.03, 67.87	1.34	0.68, 2.65
Birnbaum S.	2009	442	435	7	0	952	913	39	0	2.10	0.04, 105.89	0.40	0.18, 0.88
Carter TC.	2010	460	456	4	0	894	869	25	0	1.91	0.04, 96.15	0.34	0.12, 0.92
Paranaiba LM.	2010	177	159	18	0	126	113	13	0	0.71	0.01, 36.13	0.98	0.46, 2.05
Letra A.	2012	311	283	26	2	281	245	34	2	0.87	0.12, 6.19	0.66	0.39, 1.13
Pooled										0.75	0.27, 1.75	0.82	0.55, 1.16
Mixed ethnicity													
Velazquez JA.	2012	132	75	50	7	370	163	162	45	0.34	0.15, 0.79	0.67	0.44, 1.02

Among 5 Caucasian studies (*n* = 1704 and 2669 for NSCL/P and control), heterogeneity was not present in OR_1_ but was present for OR_2_ with an *I^2^* of 0% (Chi‐square = 0.29; d.f. = 4; *p* = 0.990) and 51.9% (Chi‐square = 8.32; d.f. = 4; *p* = 0.080), respectively. The pooled OR_1_ and OR_2_ were 0.75 (95% CI, 0.27–1.75; *p* = 0.506) and 0.82 (95% CI, 0.55–1.16; *p* = 0.262), see Figure 2C,D; indicating nonsignificant variant effects for both AA and GA genotypes when compared with GG genotypes.

Publication bias was assessed for both pooled ORs in Asian and Caucasian by funnel plot and Egger's test. There was no evidence of asymmetry of the funnel, see Supplementary Figure 1A–D. These results agreed with the Egger's tests, which indicated no evidence of asymmetry of funnels for both OR_1_ (coefficient = 1.448; *p* = 0.408) and OR_2_ (coefficient = ‐1.018; *p* = 0.358) in Asian, and OR_1_ (coefficient = 0.480; *p* = 0.261) and OR_2_ (coefficient = ‐2.763; *p* = 0.452) in Caucasian.

### rs2013162

Among the nine studies that assessed the association between rs2013162 in IRF6 and NSCL/P, four studies, three studies, and two studies were conducted in Caucasians, Asians, and in mixed ethnicity, respectively. Allele effects were pooled in Caucasian and Asian where numbers were sufficient. All studies complied with HWE with the pooled MAF A of 0.35 (95% CI,0.34–0.37) and 0.42 (95% CI, 0.35–0.50), respectively, in Caucasian, Asian of control populations. The pooled ORs of A versus C alleles were, respectively, 0.84 (95% CI, 0.76–0.93; *p* = 4.16 × 10^‐4^) and 0.99 (95% CI, 0.57–1.73; *p* = 0.974) in Caucasians and Asians (Supplementary Table 5).

**Figure 3:**
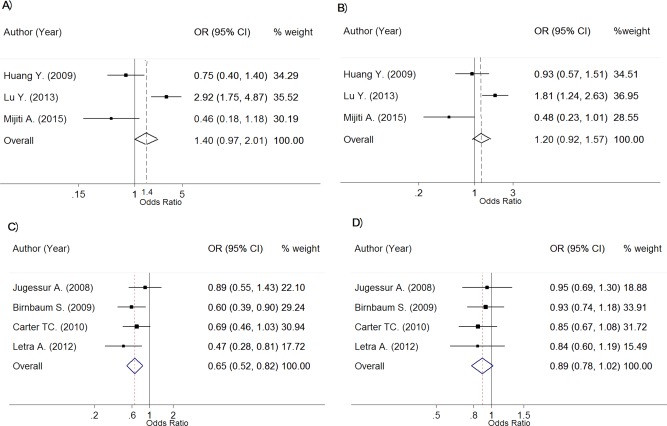
Forest plots for rs2013162 of IRF6.

The genotype frequency between NSCL/P groups of 9 studies are described in Table 3. Among four Caucasian studies (*n* = 1530 vs. 2511 for NSCL/Ps vs. controls), ORs (i.e., OR_1_ [AA vs. CC] and OR_2_ [CA vs. CC]) showed low heterogeneity for OR_1_ (Chi‐square = 3.19; d.f. = 3; *p* = 0.363; *I^2^* = 6.1%) and OR_2_ (Chi‐square = 0.57; d.f. = 3; *p* = 0.904; *I^2^* = 0%), see Figure 3A,B. The pooled OR_1_ and OR_2_ were, respectively, 0.65 (95% CI, 0.52–0.82; *p* = 1.93 × 10^‐4^) and 0.89 (95% CI, 0.78–1.02; *p* = 0.102), suggesting that the odds of NSCL/P was significantly different in AA genotype even based on Bonferroni corrected threshold, but was not significantly different in CA genotype when compared with CC genotype. The estimated lambda was 0.32 (95% CI, 0.02–0.92), which possibly indicated between a recessive to additive effect of A allele.

**Table 3:** Genotype Frequencies and Genotype Effects for IRF‐6 at SNPs 2013162 Between NSCL/P and Control Groups

		CL/P				Control							
	Year	No. of subjects	Genotype			No. of subjects	Genotype			AA/CC		CA/CC	
Author			CC	CA	AA		CC	CA	AA	OR_1_	95% CI	OR_2_	95% CI
Asian													
Huang Y.	2009	165	53	84	28	174	51	87	36	0.75	0.40, 1.40	0.93	0.57, 1.51
Lu Y.	2013	236	57	132	47	400	156	200	44	2.92	1.75, 4.87	1.81	1.24, 2.63
Mijiti A.	2015	100	44	41	15	59	16	31	12	0.46	0.18, 1,18	0.48	0.23, 1.01
Pooled										1.40	0.97, 2.01	1.20	0.92, 1.57
Caucasian													
Jugessur A.	2008	313	136	141	36	416	174	190	52	0.89	0.55, 1.43	0.95	0.69, 1.30
Birnbaum S.	2009	460	215	210	35	952	410	430	112	0.60	0.39, 0.90	0.93	0.74, 1.18
Carter TC.	2010	447	204	202	41	861	350	409	102	0.69	0.46, 1.03	0.85	0.67, 1.08
Letra A.	2012	310	143	140	27	282	110	128	44	0.47	0.28, 0.81	0.84	0.60, 1.19
Pooled										0.65	0.52, 0.82	0.89	0.78, 1.02
Mixed ethnicity													
Weatherley‐White RC.	2011	128	76	45	7	105	56	41	8	0.65	0.22, 1.88	0.81	0.47, 1.40
Bagordakis E.	2013	233	122	88	23	308	142	137	29	0.92	0.51, 1.68	0.75	0.52, 1.07

Among three Asian studies (*n* = 501 for NSCL/Ps and 633 for controls), heterogeneity was present in both OR_1_ and OR_2_ with an *I^2^* of 88.4% (Chi‐square = 17.21; d.f. = 2; *p* < 0.001) and 82.6% (Chi‐square = 11.52; d.f. = 2; *p* = 0.003), respectively. The pooled OR_1_ and OR_2_ were 1.40 (95% CI, 0.97–2.01; *p* = 0.069) and 1.20 (95% CI, 0.92–1.57; *p* = 0.172), see Figure 3C,D; indicating nonsignificant variant effects for both AA and CA genotypes when compared with CC genotypes.

Publication bias was assessed for both pooled ORs in Caucasian and Asian by funnel plot and Egger's test. Funnel plots suggested little asymmetry (Supplementary Fig. 2A–D), but the Egger's test did not confirm this. The coefficients of asymmetry was ‐2.07 (SE = 5.25; *p* = 0.731) for OR_1_ and ‐0.25 (SE = 1.64; *p* = 0.894) for OR_2_ in Caucasian, and ‐8.58 (SE = 5.45; *p* = 0.360) for OR_1_ and ‐7.41 (SE = 1.88; *p* = 0.158) for OR_2_ in Asian.

### rs642961

Eleven studies assessed effect of rs642961 polymorphism on NSCL/P. Among them, five studies, four studies, and two studies were conducted in Caucasians, Asians, and mixed ethnicity, respectively. One study (Brito et al., 2012a) did not comply with HWE and, thus, excluded from pooling. Data were sufficient for pooling in Caucasians and Asians. The pooled MAF A was 0.19 (95% CI, 0.17–0.22) and 0.17 (95% CI, 0.13–0.22) in Caucasians and Asians, respectively (Supplementary Table 6). The pooled ORs (A vs. G) were 1.50 (95% CI, 1.35–1.68, *p* = 5.11 × 10^‐13^) and 1.47 (95% CI, 1.09–1.98; *p* = 1.10 × 10^‐2^) in Caucasians and Asians, respectively.

**Figure 4:**
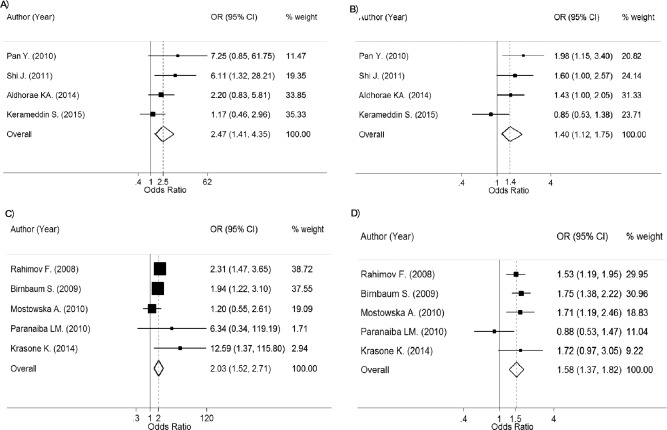
Forest plots for rs642961 of IRF6.

The genotype frequencies between NSCL/P and control groups are described in Table 4. Genotype effects were sufficient for pooling in Caucasians and Asians. Among 5 Caucasians studies (*n* = 1244 vs. 3069 for NSCL/Ps and controls), the OR_1_ (AA vs. GG) and OR_2_ (GA vs. GG) were estimated with low heterogeneity for OR_1_ (Chi‐square = 5.31; d.f. = 4; *p* = 0.257; *I^2^* = 24.6%) and moderate for OR_2_ (Chi‐square = 6.08; d.f. = 4; *p* = 0.194; *I^2^* = 34.2%). The mixed‐effect logit model yielded pooled OR_1_ and OR_2_ of 2.03 (95% CI, 1.52–2.71; *p* = 1.81 × 10^‐6^) and 1.58 (95% CI, 1.37–1.82; *p* = 4.59 × 10^‐10^), which suggested that persons with AA and GA genotypes were 2.03 and 1.58 times significantly higher risk of NSCL/P than persons with GG genotype (Fig. 4A,B). These effects were still significant based on Bonferroni corrected threshold. The estimated lambda value was 0.62 (95% CI, 0.17–0.96) suggesting a potential mode of effect between recessive to additive effect.

**Table 4:** Genotype Frequencies and Genotype Effects for IRF‐6 at SNPs 642961 Between NSCL/P and Control Groups

		CL/P				Control							
	Year	No. of subjects	Genotype			No. of subjects	Genotype			AA/GG		GA/GG	
Author			GG	GA	AA		GG	GA	AA	OR_1_	95% CI	OR_2_	95% CI
Asian													
Pan Y.	2010	127	67	54	6	115	81	33	1	7.25	0.85, 61.75	1.98	1.15, 3.40
Shi J.	2011	173	100	62	11	156	111	43	2	6.11	1.32, 28.21	1.60	1.00, 2.57
Aldhorae KA.	2014	242	159	74	9	420	311	101	8	2.20	0.83, 5.81	1.43	1.00, 2.05
Kerameddin S.	2015	150	88	51	11	150	84	57	9	1.17	0.46, 2.96	0.85	0.53, 1.38
Pooled										2.47	1.41, 4.35	1.40	1.12, 1.75
Caucasian													
Rahimov F.	2008	368	180	155	33	1244	757	427	60	2.31	1.47, 3.65	1.53	1.19, 1.95
Birnbaum S.	2009	460	237	189	34	951	622	283	46	1.94	1.22, 3.10	1.75	1.38, 2.22
Mostowska A.	2010	165	82	74	9	565	349	184	32	1.20	0.55, 2.61	1.71	1.19, 2.46
Paranaiba LM.	2010	177	128	45	4	126	90	36	0	6.34	0.34, 119.19	0.88	0.53, 1.47
Krasone K.	2014	74	41	29	4	183	129	53	1	12.59	1.37, 115.80	1.72	0.97, 3.05
Pooled										2.03	1.52, 2.71	1.58	1.37, 1.82
Mixed ethnicity													
Brito LA.	2012	471	320	123	28	391	285	90	16	1.56	0.83, 2.94	1.22	0.89, 1.67
do Rego Borges A.	2015	293	214	73	6	352	282	63	7	1.13	0.37, 3.41	1.53	1.04, 2.24

Among four Asians studies (*n* = 300 vs. 271 for NSCL/Ps and controls), the OR_1_ (AA vs. GG) and OR_2_ (GA vs. GG) were estimated with moderate heterogeneity for OR_1_ (Chi‐square = 4.77; d.f. = 3; *p* = 0.189; *I^2^* = 37.1%) and OR_2_ (Chi‐square = 5.93; d.f. = 3; *p* = 0.115; *I^2^* = 49.4%). The mixed‐effect logit model yielded pooled OR_1_ and OR_2_ of 2.47 (95% CI, 1.41–4.35; *p* = 1.65 × 10^‐3^) and 1.40 (95% CI, 1.12–1.75; *p* = 3.02 × 10^‐3^), which suggested that persons with AA and GA genotypes were 2.47 and 1.40 times significantly higher risk of NSCL/P than persons with GG genotype (Fig. 4C,D). However, only OR_1_ was still significant based on Bonferroni corrected threshold. The estimated lambda value was 0.62 (95% CI, 0.17–0.96) suggesting a potential mode of variant effect could be from a recessive to additive mode.

No evidence of reporting bias was seen with either OR_1_ or OR_2_ suggested from Egger tests (in Caucasian: coefficient = 0.98; SE = 0.98; *p* = 0.391 for OR_1_ and coefficient = ‐1.61; SE = 1.68; *p* = 0.409 for OR_2_; in Asian: coefficient = 3.14; SE = 1.22; *p* = 0.124 for OR_1_ and coefficient = 0.66; SE = 5.33; *p* = 0.913 for OR_2_) and funnel plots (Supplementary Fig. 3A–D).

### rs987525

Allele frequencies of 12 studies assessed effect of rs987525 polymorphism on NSCL/P and all observed HWE (Supplementary Table 7). The MAF A in controls were 0.15 (95% CI, 0.00–0.31) in three Asian studies, and 0.20 (95% CI, 0.17–0.22) in four Caucasian studies, and 0.27 (95% CI, 0.17–0.38) in five mixed ethnicity. The estimated ORs for allele A versus C in these corresponding ethnicities were 1.48 (95% CI, 1.21–1.81; *p* = 1.21 × 10^‐4^), and 2.20 (95% CI, 1.96–2.46; *p* < 1.00 × 10^‐12^), and 1.21 (95% CI, 1.09–1.36; *p* = 7.5 × 10^‐4^), respectively.

**Figure 5:**
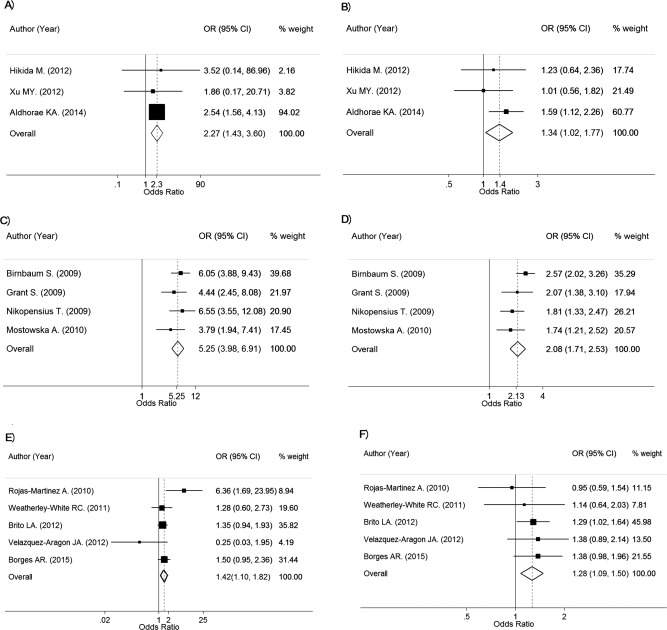
Forest plots for rs987525 of 8q24.

Among three Asian studies (*n* = 625 in NSCL/P and 811 in controls), the pooled OR_1_ (AA vs. CC) and OR_2_ (CA vs. CC) were, respectively, 2.27 (95% CI, 1.43–3.60; *p* = 4.71 × 10^‐4^) and 1.34 (95% CI, 1.02–1.77; *p* = 3.71 × 10^‐2^), with no heterogeneity for OR_1_ (Chi‐square = 0.10; d.f. = 2; *p* = 0.950; *I^2^* = 0%) and OR_2_ (Chi‐square = 1.83; d.f. = 2; *p* = 0.401; *I^2^* = 0%) (Table 5; Fig. 5A,B). This indicates that those with AA and CA genotypes were 2.27 and 1.34 times significantly, based on Bonferroni corrected threshold, more likely to have NSCL/P than those with the CC genotype. The estimated lambda was 0.45 (95% CI, 0.27–0.67), indicating an additive model was most likely.

**Table 5:** Genotype Frequencies and Genotype Effects for 8q24 at SNPs 987525 Between NSCL/P and Control Groups

		CL/P				Control							
	Year	No. of subjects	Genotype			No. of subjects	Genotype			AA/CC		CA/CC	
Author			CC	CA	AA		CC	CA	AA	OR_1_	95% CI	OR_2_	95% CI
Asian													
Hikida M.	2012	167	145	21	1	190	170	20	0	3.52	0.14, 86.96	1.23	0.64, 2.36
Xu MY.	2012	216	188	26	2	200	175	24	1	1.86	0.17, 20.71	1.01	0.56, 1.82
Aldhorae KA.	2014	242	76	119	47	421	189	186	46	2.54	1.56, 4.13	1.59	1.12, 2.26
Pooled										2.27	1.43, 3.60	1.34	1.02, 1.77
Caucasian													
Birnbaum S.	2009	462	172	228	62	952	604	312	36	6.05	3.88, 9.43	2.57	2.02, 3.26
Grant S.	2009	111	45	51	15	5951	3667	2009	275	4.45	2.45, 8.08	2.07	1.38, 3.10
Nikopensius T.	2009	217	114	82	21	1267	889	353	25	6.55	3.55, 12.08	1.81	1.33, 2.47
Mostowska A.	2010	165	77	70	18	565	357	186	22	3.79	1.94, 7.41	1.75	1.21, 2.52
Pooled										5.25	3.98, 6.91	2.13	1.82, 2.49
Mixed ethnicity													
Rojas‐Martinez A.	2010	149	109	31	9	303	231	69	3	6.36	1.69, 23.95	0.95	0.59, 1.54
Weatherley‐White RC.	2011	129	42	63	24	105	38	50	17	1.28	0.60, 2.73	1.14	0.64, 2.03
Brito LA.	2012	667	278	302	87	589	284	239	66	1.35	0.94, 1.93	1.29	1.02, 1.64
Velazquez JA.	2012	132	89	42	1	370	267	91	12	0.25	0.03, 1.95	1.39	0.89, 2.14
do Rego Borges A.	2015	293	94	143	56	352	141	155	56	1.50	0.95, 2.36	1.38	0.98, 1.96
Pooled										1.42	1.10, 1.82	1.28	1.09, 1.50

Among four Caucasian studies (*n* = 955 in NSCL/P and 8735 in controls), the pooled OR_1_ (AA vs. CC) and OR_2_ (CA vs. CC) were, respectively, 5.25 (95% CI, 3.98–6.91; *p* < 1.00 × 10^‐12^) and 2.13 (95% CI, 1.82–2.49; *p* < 1.00 × 10^‐12^), with no heterogeneity for OR_1_ (Chi‐square = 2.09; d.f. = 3; *p* = 0.553; *I^2^* = 0%) and moderate heterogeneity for OR_2_ (Chi‐square = 4.50; d.f. = 3; *p* = 0.212; *I^2^* = 33.3%) (Table 5; Fig. 5C,D). This indicates that those with AA and CA genotypes were 5.25 and 2.13 times significantly, based on Bonferroni corrected threshold, more likely to have NSCL/P than those with the CC genotype. The estimated lambda was 0.45 (95% CI, 0.27–0.67), indicating an additive model was most likely.

Among five studies with mixed ethnicity (*n* = 1370 in NSCL/P and 1719 in controls), the corresponding pooled ORs were 1.42 (95% CI, 1.10–1.82; *p* = 6.10 × 10^‐3^) and 1.28 (95% CI, 1.09–1.50; *p* = 2.47 × 10^‐3^) with moderate heterogeneity for OR_1_ (Chi‐square = 7.87; d.f. = 4; *p* = 0.096; *I^2^* = 49.2%) and no evidence of heterogeneity for OR_2_ (Chi‐square = 1.92; d.f. = 4; *p* = 0.751; *I^2^* = 0%) (Fig. 5F,G). These two ORs were not significant based on Bonferroni corrected threshold. The estimated lambda 0.68; 95% CI, 0–1), which could possibly be between additive to dominant mode.

There was no evidence of reporting bias (in Asian: Egger coefficient = ‐0.02; SE = 0.28; *p* = 0.951 for OR_1_ and coefficient = ‐2.50; SE = 1.29; *p* = 0.303 for OR_2_; in Caucasian: coefficient = ‐2.76; SE = 2.48; *p* = 0.380 for OR_1_ and coefficient = ‐3.93; SE = 2.47; *p* = 0.252 for OR_2_; in mixed ethnicity: coefficient = 0.10; SE = 1.45; *p* = 0.951 for OR_1_ and coefficient = ‐0.93; SE = 1.00; *p* = 0.422 for OR_2_; see Funnel plots in Supplementary Figure 4A–F.

## Discussion

We conducted a systematic review and meta‐analysis to assess genetic effects of polymorphisms in *IRF6* and 8q24 region on NSCL/P. A total of 9 to 15 studies were included in the pooling for three polymorphisms (i.e., rs2235371, rs2013162, rs642961) for *IRF6* and for rs987525 in 8q24. The MAF for these corresponding polymorphisms were similar across ethnicities except for rs2235371 where the MAF was common in Asians but rare in Caucasians, whereas MAF for rs987525 was common in Caucasians but less common in Asians. Because the MAF of rs2235371 was different between ethnicities, the genotype effect for this SNP was different. All polymorphisms were significantly associated with NSCL/P in some ethnicities. A significant protective effect of rs2235371 in Asians and effect of rs2013162 in Caucasians were suggested, that is, carrying one copy of the A allele for both polymorphisms lowered the odds of NSCL/P compared with G and C alleles, respectively. In addition, our findings indicated risk effects of minor alleles for rs642961 and rs987525.

Our findings are consistent with previous human GWAS studies (Birnbaum et al., 2009b; Grant et al., 2009; Beaty et al., 2010; Ludwig et al., 2012). In addition, our findings are also consistent with the previous meta‐analysis of genetic association studies by Wang et al. (2012), that included 10, 6, and 8 studies in the pooling of rs2235371 and rs642961 for *IRF6*, and rs987525 for 8q24, respectively. We confirmed the previous findings by expanding to a larger number of included genetic association studies (i.e., 15, 11, and 12 studies for these corresponding polymorphisms) and applying Bonferroni correction for multiple tests. Although each polymorphism was considered separately in meta‐analysis for genetic association studies, this type of study had been used to replicate and confirm GWAS. Not only allele effects were estimated, but also pooled MAF and genotype effects by ethnicity additionally provided from previous evidences.

Formation of the lip and palate in human embryos starts at the fourth week and is complete by the twelfth week after fertilization. This process occurs by migration of the ectoderm, and formation of frontal, medial and lateral nasal processes, which then fuse together to form the normal lips and nose (Shkoukani et al., 2013). A normal palate also occurs after complete fusion of the primary and secondary palates; failure of fusion causes separation of the lip/s and/or palate, that is, cleft lip and cleft palate, respectively. Although the function of the *IRF6* gene on the cleft lip formation is unclear, evidence has supported an effect of this gene on other birth malformations including Van der Woude syndrome and popliteal pterygium syndrome (Kondo et al., 2002; Kayano et al., 2003; Shotelersuk et al., 2003; Gatta et al., 2004; Ghassibé et al., 2004; Item et al., 2004). High IRF6 mRNA levels are also seen at the medial edge of the fusing palate (Kondo et al., 2002).

In addition, evidence from animal studies also supports the role of IRF6 on NSCL/P. Animal models have shown abnormalities of epithelial differentiation in mice with a mutation of *IRF6* (Iwata et al., 2013). This supports a role for IRF6 in the formation and maintenance of the periderm, and spatiotemporal regulation for appropriate palatal adhesion (Richardson et al., 2009). Another study on mice with mutation of IRF6 showed abnormal skin, limb, and craniofacial development, which suggests a role for IRF6 in keratinocyte proliferation and differentiation of face and limb (Ingraham et al., 2006). The role of the 8q24 region on NSCL/P is still unclear but it might be involved in maintenance of an undifferentiated state in human acute myeloblastic leukemia cells (HL60) (Hirano et al., 2008), control of Myc expression (Uslu et al., 2014), or cranial facial enhancers (Attanasio et al., 2013).

The etiology of NSCL/P is still unclear but evidence suggests that both genetic and nongenetic factors play a role (Dixon et al., 2011; Shkoukani et al., 2013). Environmental factors may also play a role in NSCL/P. These factors may be independent or they may be modified by genes such as 5, 10‐methylenetetrahydrofolate reductase and folate (Bufalino et al., 2010). Evidence from cohort studies suggested that oral folate supplements before or during the periconception period might be able to reduce risk of NSCL/P (van Rooij et al., 2004; Kelly et al., 2012; Li et al., 2012); this is being confirmed by a randomized controlled trial (Wehby et al., 2012). Other factors associated with NSCL/P include alcohol consumption (Lieff et al., 1999; DeRoo et al., 2008), gestational diabetes (Correa et al., 2008), maternal age>40 years (Herkrath et al., 2012), and zinc deficiency (Warkany and Petering, 1972).

Our results also suggested that the *IRF6* ((i.e., rs2235371, rs2013162, rs642961) and 8q24 (rs987525) SNPs contributed to NSCL/P, in which the rs987525 had highest contribution. This might be comparable to the effect of folate with an estimated PAR approximately 18% (van Rooij et al., 2004; Kelly et al., 2012). For public health prevention, screening these genes and other genes identified by previous meta‐analyses (e.g., *VAX1*, Figueiredo et al., 2014; or bone morphogenetic protein 4, Hu et al., 2015), and combining them with environmental factors (e.g., folate, alcohol, etc.) may lead to a useful risk score for prediction of NSCL/P occurrence (Seddon et al., 2009).

Our study has some strengths. We attempted to pool variant effects on NSCL/P separately by ethnicity if data were available. Prevalence of MAFs for each study polymorphism was pooled across studies and ethnicity. Mode of variant effect was estimated using a mixed‐effect logistic regression (Thakkinstian et al., 2005a) to capture lambda (Minelli et al., 2005). However, our study also has some limitations. First, given that we worked on summary data, we could not control for confounding effects, although the major source of confounding for genetic studies is population stratification and we summarized results by ethnic group. Second, we could only identify association between genes and NSCL/P, and we do not know whether the genes directly affect NSCL/P or whether their effects might be exerted through other environmental factors, such as folate. Exploring this would require individual patient data where data for folate and other co‐variables are also available. Third, we could not identify which polymorphism is the disease gene among those three polymorphisms in *IRF6* gene because evidences showed linkage disequilibrium between rs2235371 and rs2013162 in Mexican (Ibarra‐Arce et al., 2015) and Chinese (Mijiti et al., 2015) population (r^2^ = 0.39 and 0.25, respectively) but not for rs642961 (Pan et al., 2010) (r^2^ = 0.07). To determine this required raw data for performing a haplotype analysis. Some variant effects were heterogeneous (i.e., varied) across studies; we could explore for a cause of heterogeneity by fitting percent male in a meta‐regression, but this was not detected (data were not shown). There might be other characteristics that cause heterogeneity, but those data were not available. Finally, there might be gene to gene interactions, but summary data do not allow us to explore this.

### Conclusions

All polymorphisms, rs2235371, rs2013162, and rs642961 at *IRF6* and rs987525 in 8q24 were significantly associated with NSCL/P. However, after applying Bonferroni corrected‐thresholds, variant effects remained significant, except in mixed ethnicity. We show robust variant effects in NSCL/P. Including them with other risk factors and other genes in a risk prediction model of NSCL/P might be useful to improve prediction of NSCL/P occurrence.

## Supporting information

Additional Supporting information may be found in the online version of this article.

Supplement Figure 1. Funnel plots for rs2235371 of IRF6.

Supplement Figure 2. Funnel plots for rs2013162 of IRF6.

Supplement Figure 3. Funnel plots for rs642961 of IRF6.

Supplement Figure 4. Funnel plots for rs987525 of 8q24.

Supplement Tables.
