# Supplementary figures and images for: Association between IRF6 and 8q24 polymorphisms and nonsyndromic cleft lip with or without cleft palate: Systematic review and meta‐analysis
Source: Birth Defects Res A Clin Mol Teratol. 2016 Aug 11;106(9):773–88. doi: 10.1002/bdra.23540 (PMC5095821; doi:10.1002/bdra.23540)

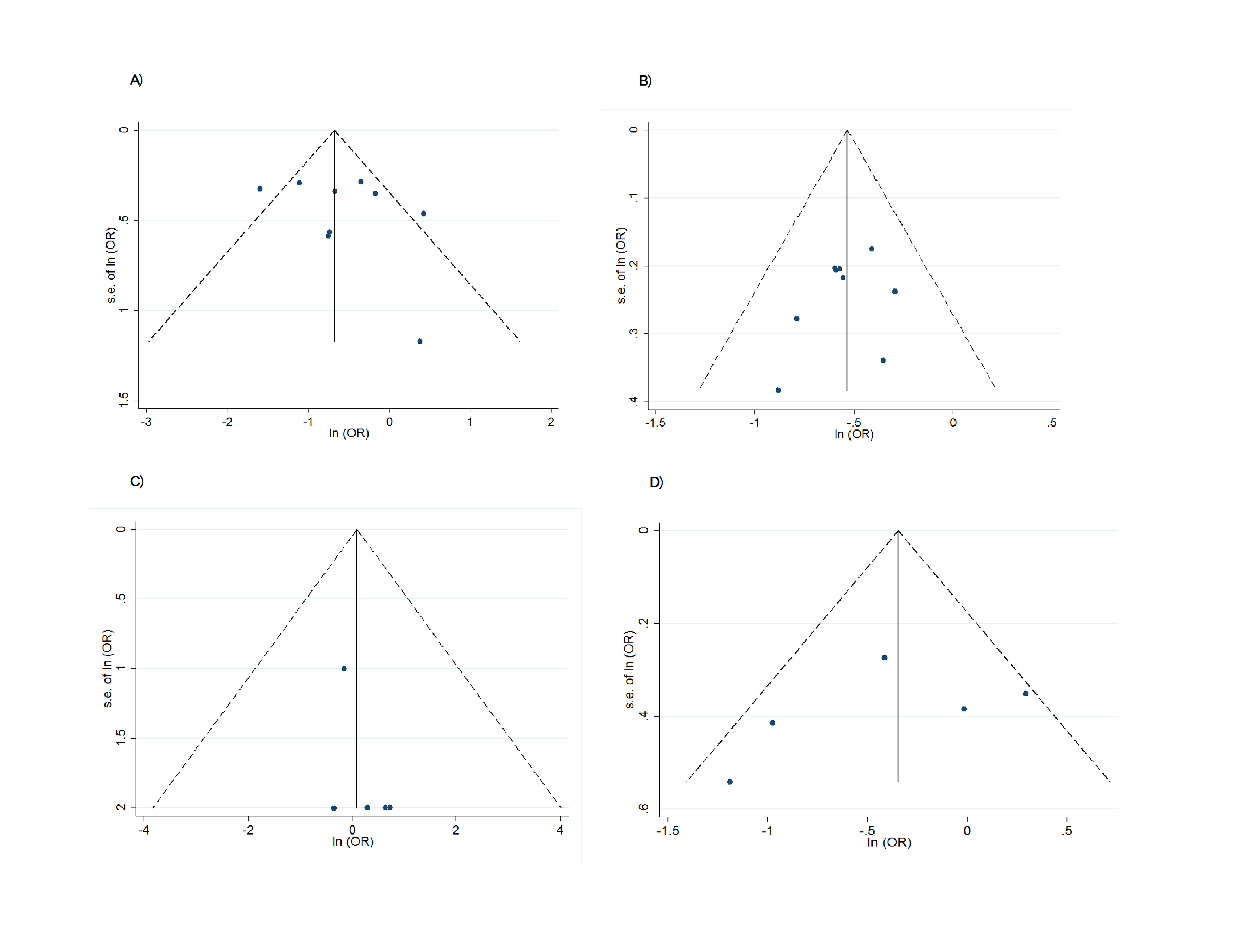

Supplement: Supplementary file 1 — Supplement Figure 1. Funnel plots for rs2235371 of IRF6. [file BDRA-106-773-s001.tif]

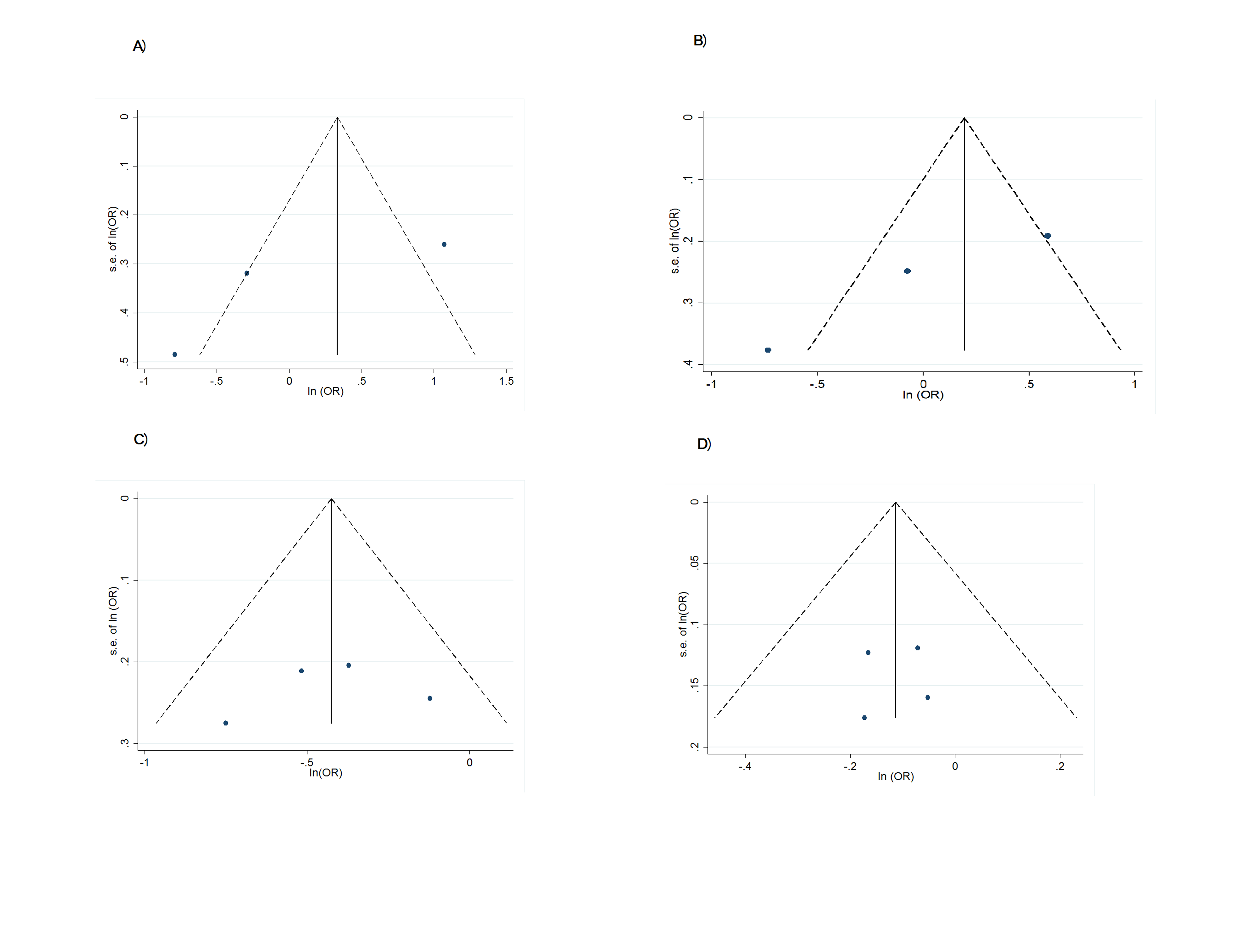

Supplement: Supplementary file 2 — Supplement Figure 2. Funnel plots for rs2013162 of IRF6. [file BDRA-106-773-s002.tif]

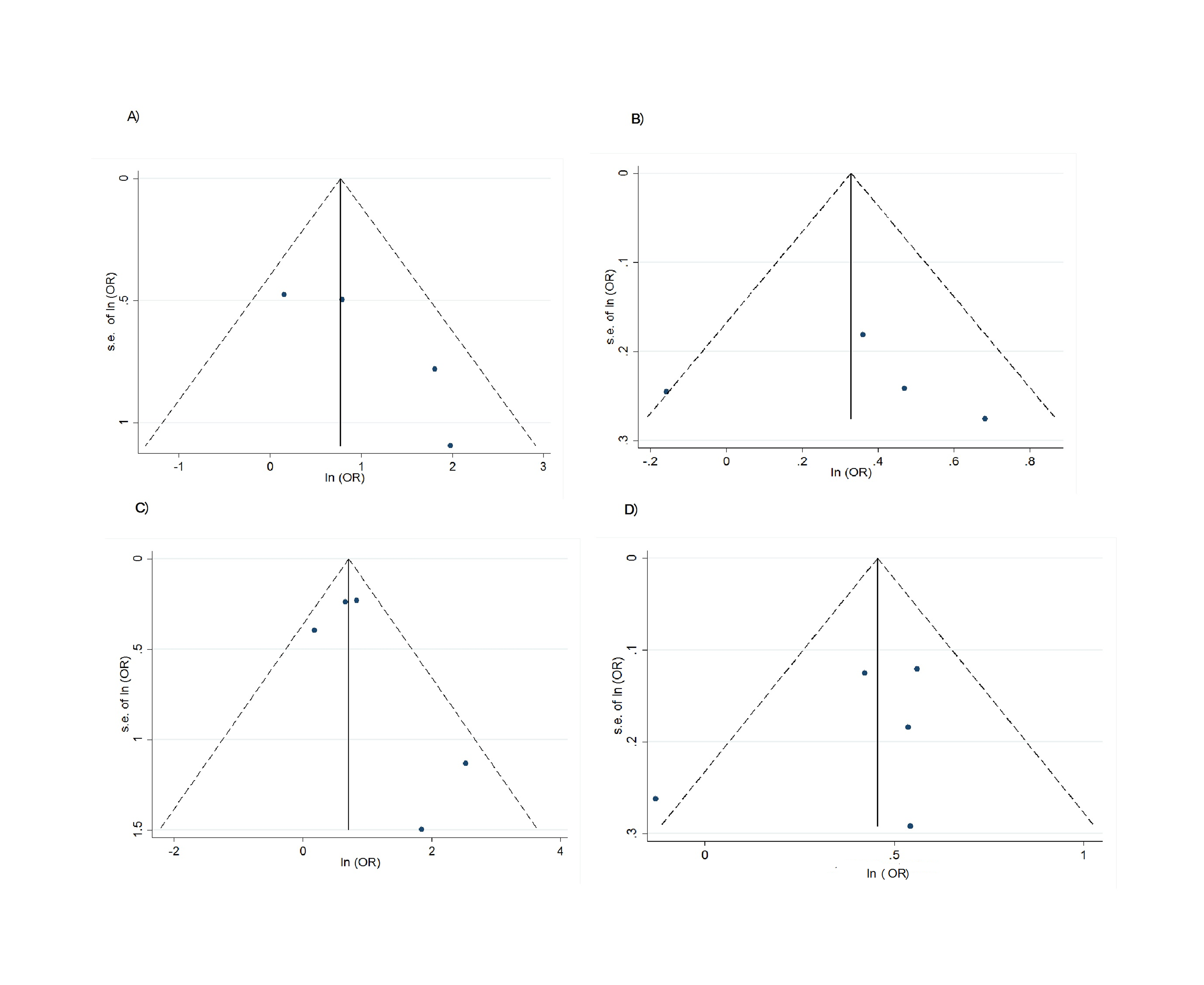

Supplement: Supplementary file 3 — Supplement Figure 3. Funnel plots for rs642961 of IRF6. [file BDRA-106-773-s003.tif]

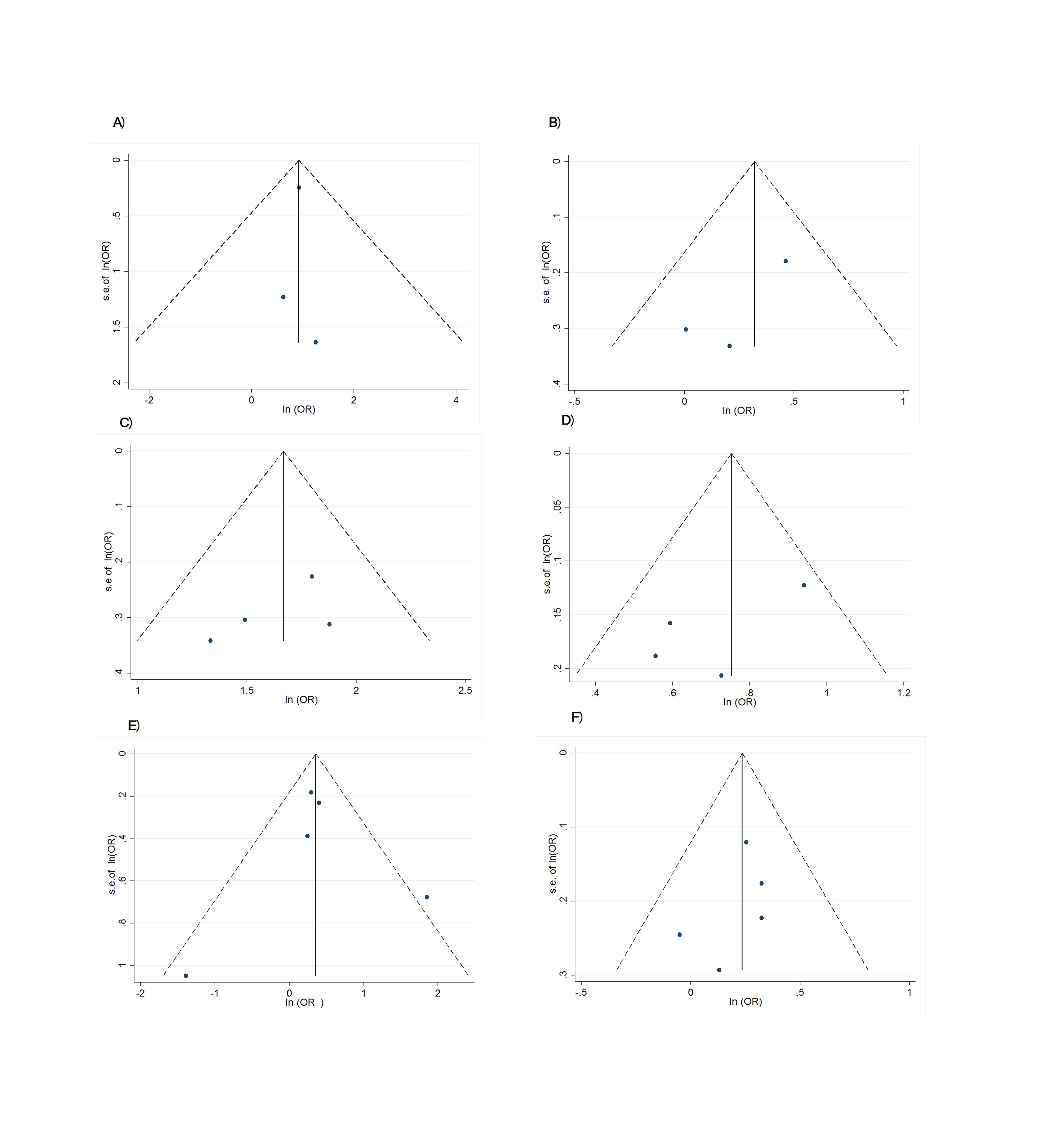

Supplement: Supplementary file 4 — Supplement Figure 4. Funnel plots for rs987525 of 8q24. [file BDRA-106-773-s004.tif]
