# Supplementary material for: Association between IRF6 and 8q24 polymorphisms and nonsyndromic cleft lip with or without cleft palate: Systematic review and meta‐analysis
Source: Birth Defects Res A Clin Mol Teratol. 2016 Aug 11;106(9):773–88. doi: 10.1002/bdra.23540 (PMC5095821; doi:10.1002/bdra.23540)
Supplement: Supplementary file 5 — Supplement Tables. [file BDRA-106-773-s005.docx]

Supplement Table 1

Search Strategy

| Search | Query | Item |
| --- | --- | --- |
| #31 | Search (#18) AND #30 | 307 |
| #30 | Search ((((((#19) OR #20) OR #21) OR #22) OR #23) OR #24) OR #25 | 841018 |
| #25 | Search "nonsyndromic cleft" | 454 |
| #24 | Search "cleft palate" | 20944 |
| #23 | Search CPO | 1273 |
| #22 | Search CP | 55998 |
| #21 | Search CL | 765158 |
| #20 | Search cleft lip | 16032 |
| #19 | Search NSCL/P | 149 |
| #18 | Search (#9) AND #17 | 4252 |
| #17 | Search ((((((#10) OR #11) OR #12) OR #13) OR #14) OR #15) OR #16 | 2245470 |
| #16 | Search genes | 1192494 |
| #15 | Search SNPs | 98317 |
| #14 | Search SNP | 40945 |
| #13 | Search allele | 212595 |
| #12 | Search gene | 2144196 |
| #11 | Search polymorphisms | 263580 |
| #10 | Search polymorphism | 260468 |
| #9 | Search ((((#4) OR #5) OR #6) OR #7) OR #8 | 6174 |
| #8 | Search 8q24 | 1613 |
| #7 | Search "interferon regulation factor 6" | 4340 |
| #6 | Search "interferon regulatory factor 6" | 105 |
| #5 | Search irf-6 | 10 |
| #4 | Search irf6 | 260 |

Supplement Table 2

Describe a total number of tests

| No. | Polymorphism | Ethnicity |
| --- | --- | --- |
|  | rs2235371 |  |
| 1 | OR (A vs G) | Asian |
| 2 | OR1 (AA vs GG) | Asian |
| 3 | OR2 (AG vs GG) | Asian |
| 4 | OR (A vs G) | Caucasian |
| 5 | OR1 (AA vs GG) | Caucasian |
| 6 | OR2 (AG vs GG) | Caucasian |
|  | rs2013162 |  |
| 7 | OR (A vs C) | Asian |
| 8 | OR1 (AA vs CC) | Asian |
| 9 | OR2 (AA vs CC) | Asian |
| 10 | OR (A vs C) | Caucasian |
| 11 | OR1 (AA vs CC) | Caucasian |
| 12 | OR2 (AC vs CC) | Caucasian |
|  | rs642961 |  |
| 13 | OR (A vs G) | Asian |
| 14 | OR1 (AA vs GG) | Asian |
| 15 | OR2 (AG vs GG) | Asian |
| 16 | OR (A vs G) | Caucasian |
| 17 | OR1 (AA vs GG) | Caucasian |
| 18 | OR2 (AG vs GG) | Caucasian |
|  | rs987525 |  |
| 19 | OR (A vs C) | Asian |
| 20 | OR1 (AA vs CC) | Asian |
| 21 | OR2 (AC vs CC) | Asian |
| 22 | OR (A vs C) | Caucasian |
| 23 | OR1 (AA vs CC) | Caucasian |
| 24 | OR2 (AC vs CC) | Caucasian |
| 25 | OR (A vs C) | Mixed |
| 26 | OR1 (AA vs CC) | Mixed |
| 27 | OR2 (AC vs CC) | Mixed |

OR, odds ratio

Supplementary Table 3

Risk of Bias Assessments

| Author | Year | Ascertainment of NSCL/P | Ascertainment of control | Quality control for genotyping | Population stratification | Confounding bias | Selective outcome report | HWE |
| --- | --- | --- | --- | --- | --- | --- | --- | --- |
| Srichomthong C. | 2005 | yes | yes | unclear | yes | no | yes | yes |
| Jugessur A. | 2008 | yes | yes | unclear | yes | no | yes | yes |
| Rahimov F. | 2008 | yes | yes | yes | yes | no | yes | no |
| Ali A. | 2009 | yes | yes | unclear | yes | yes | yes | yes |
| Birnbaum S. | 2009 | yes | no | unclear | yes | yes | yes | yes |
| Birnbaum S. | 2009 | yes | yes | yes | yes | yes | no | no |
| Grant S. | 2009 | yes | no | yes | yes | no | yes | yes |
| Huang Y. | 2009 | yes | yes | yes | yes | no | yes | yes |
| Nikopensius T. | 2009 | yes | yes | yes | yes | yes | yes | yes |
| Tang W. | 2009 | yes | yes | yes | yes | no | yes | yes |
| Carter TC. | 2010 | yes | yes | yes | yes | no | yes | yes |
| Mostowska A. | 2010 | yes | yes | yes | yes | yes | yes | yes |
| Pan Y. | 2010 | yes | yes | yes | yes | yes | yes | yes |
| Paranaiba L. | 2010 | yes | yes | yes | yes | yes | yes | yes |
| Rojas-Martinez A. | 2010 | yes | yes | yes | yes | no | yes | yes |
| Shi J. | 2011 | yes | yes | unclear | yes | no | yes | yes |
| Weatherley-White R. | 2011 | yes | yes | unclear | yes | yes | yes | yes |
| Brito L. (a) | 2012 | yes | no | yes | yes | no | yes | yes |
| Brito L. (b) | 2012 | yes | no | unclear | yes | no | yes | yes |

Supplementary Table 3

Risk of Bias Assessments (continuous)

| Author | Year | Ascertainment of NSCL/P | Ascertainment of control | Quality control for genotyping | Population stratification | Confounding bias | Selective outcome report | HWE |
| --- | --- | --- | --- | --- | --- | --- | --- | --- |
| Hikida M. | 2012 | yes | yes | yes | yes | no | yes | yes |
| Letra A. | 2012 | yes | yes | yes | yes | no | yes | yes |
| Velazquez JA. | 2012 | yes | yes | yes | yes | no | yes | yes |
| Xu M. | 2012 | yes | yes | yes | yes | no | yes | yes |
| Bagordakis E. | 2013 | yes | yes | yes | yes | no | yes | yes |
| Lu Y. | 2013 | yes | yes | yes | yes | no | yes | yes |
| Song T. | 2013 | yes | yes | yes | yes | no | yes | yes |
| Aldhorae KA. | 2014 | yes | yes | yes | yes | no | yes | yes |
| Krasone K. | 2014 | yes | yes | yes | yes | no | yes | yes |
| Do Rego Borges A. | 2015 | yes | yes | yes | yes | no | yes | yes |
| Kerameddin S. | 2015 | yes | yes | yes | yes | no | yes | yes |
| Mijiti A. | 2015 | yes | yes | yes | yes | no | yes | yes |

Supplementary Table 4

Allele Frequencies and Estimated Pooled Prevalence of Major and Minor Alleles for IRF-6 at SNPs 2235371 by Ethnicity and Disease Groups

| Author | Year | CL/P | | | | | Control | | | | | A/G | | HWE |
| --- | --- | --- | --- | --- | --- | --- | --- | --- | --- | --- | --- | --- | --- | --- |
|  |  | No. of | G allele | | A allele | | No. of | G allele | | A allele | | OR | 95% CI | P value |
|  |  | allele | Freq. | Prev. | Freq. | Prev. | allele | Freq. | Prev. | Freq. | Prev. |  |  |  |
| **Asian** |  |  |  |  |  |  |  |  |  |  |  |  | |  |
| Srichomthong C. | 2005 | 384 | 258 | 0.67 | 126 | 0.33 | 556 | 337 | 0.61 | 219 | 0.39 | 0.75 | 0.57, 0.99 | 0.593 |
| Ali A. | 2009 | 646 | 569 | 0.88 | 77 | 0.12 | 428 | 348 | 0.81 | 80 | 0.19 | 0.59 | 0.42, 0.83 | 0.830 |
| Huang Y. | 2009 | 514 | 372 | 0.72 | 142 | 0.28 | 348 | 193 | 0.55 | 155 | 0.45 | 0.48 | 0.36, 0.63 | 0.285 |
| Tang W. | 2009 | 132 | 91 | 0.69 | 41 | 0.31 | 192 | 118 | 0.61 | 74 | 0.39 | 0.72 | 0.45, 1.15 | 0.330 |
| Pan Y. | 2010 | 254 | 178 | 0.70 | 76 | 0.30 | 230 | 154 | 0.67 | 76 | 0.33 | 0.87 | 0.59, 1.27 | 0.055 |
| Shi J. | 2011 | 346 | 233 | 0.67 | 113 | 0.33 | 308 | 197 | 0.64 | 111 | 0.36 | 0.86 | 0.62, 1.19 | 1.000 |
| Lu Y. | 2013 | 472 | 340 | 0.72 | 132 | 0.28 | 800 | 484 | 0.61 | 316 | 0.40 | 0.60 | 0.47, 0.76 | 0.242 |
| Song T. | 2013 | 406 | 300 | 0.74 | 106 | 0.26 | 452 | 292 | 0.65 | 160 | 0.35 | 0.65 | 0.48, 0.87 | 0.669 |
| Mijiti A. | 2015 | 200 | 177 | 0.89 | 23 | 0.12 | 120 | 98 | 0.82 | 22 | 0.18 | 0.58 | 0.31, 1.09 | 0.772 |
| Pooled |  |  | 0.74 (0.68, 0.81) | | 0.26 (0.19, 0.32) | |  | 0.66 (0.60, 0.72) | | 0.34 (0.28, 0.40) | | 0.66 | 0.58, 0.75 |  |
| **Caucasian** |  |  |  |  |  |  |  |  |  |  |  |  | |  |
| Jugessur A. | 2008 | 628 | 611 | 0.97 | 17 | 0.03 | 832 | 815 | 0.98 | 17 | 0.02 | 1.33 | 0.68, 2.63 | 1.000 |
| Birnbaum S. | 2009 | 884 | 877 | 0.99 | 7 | 0.01 | 1904 | 1865 | 0.98 | 39 | 0.02 | 0.38 | 0.17, 0.86 | 1.000 |
| Carter TC. | 2010 | 920 | 916 | 1.00 | 4 | 0.00 | 1788 | 1763 | 0.99 | 25 | 0.01 | 0.31 | 0.11, 0.89 | 1.000 |
| Letra A. | 2012 | 622 | 592 | 0.95 | 30 | 0.05 | 562 | 524 | 0.93 | 38 | 0.07 | 0.70 | 0.43, 1.14 | 0.362 |
| Paranaiba LM. | 2010 | 354 | 336 | 0.95 | 18 | 0.05 | 252 | 239 | 0.95 | 13 | 0.05 | 0.99 | 0.47, 2.05 | 1.000 |
| Pooled |  |  | 0.98 (0.96, 0.99) | | 0.02 (0.01, 0.04) | |  | 0.97 (0.96, 0.98) | | 0.03 (0.02, 0.04) | | 0.69 | 0.43, 1.12 |  |
| **Mixed ethnicity** |  |  |  | |  | |  |  | |  | |  | |  |
| Velazquez JA. | 2012 | 264 | 200 | 0.76 | 64 | 0.24 | 740 | 488 | 0.66 | 252 | 0.34 | 0.62 | 0.45, 0.85 | 0.628 |

Supplementary Table 5

Allele Frequencies and Estimated Pooled Prevalence of Major and Minor Alleles for IRF-6 at SNPs 2013162 by Ethnicity and Disease Groups

| Author | Year | CL/P | | | | | Control | | | | | A/C | | HWE |
| --- | --- | --- | --- | --- | --- | --- | --- | --- | --- | --- | --- | --- | --- | --- |
|  |  | No. of alleles | C allele | | A allele | | No. of alleles | C allele | | A allele | | OR | 95% CI | p value |
|  |  |  | Freq. | Prev. | Freq. | Prev. |  | Freq. | Prev. | Freq. | Prev. |  |  |  |
| **Asian** |  |  |  |  |  |  |  |  |  |  |  |  | |  |
| Huang Y. | 2009 | 330 | 190 | 0.58 | 140 | 0.42 | 348 | 189 | 0.54 | 159 | 0.46 | 0.88 | 0.65, 1.19 | 0.921 |
| Lu Y. | 2013 | 472 | 246 | 0.52 | 226 | 0.48 | 800 | 512 | 0.64 | 288 | 0.36 | 1.63 | 1.30, 2.06 | 0.089 |
| Mijiti A. | 2015 | 200 | 129 | 0.65 | 71 | 0.36 | 118 | 63 | 0.53 | 55 | 0.47 | 0.63 | 0.40, 1.00 | 0.796 |
| Pooled |  |  | 0.58 (0.51, 0.65) | | 0.42 (0.36, 0.49) | |  | 0.58 (0.50, 0.66) | | 0.42 (0.35, 0.50) | | 0.99 (0.57, 1.73) | |  |
| **Caucasian** |  |  |  |  |  |  |  |  |  |  |  |  |  |  |
| Jugessur A. | 2008 | 626 | 413 | 0.66 | 213 | 0.34 | 832 | 538 | 0.65 | 294 | 0.35 | 0.94 | 0.76, 1.17 | 0.991 |
| Birnbaum S. | 2009 | 920 | 640 | 0.70 | 280 | 0.30 | 1904 | 1250 | 0.66 | 654 | 0.34 | 0.84 | 0.71, 0.99 | 0.963 |
| Carter TC. | 2010 | 894 | 610 | 0.68 | 284 | 0.32 | 1722 | 1109 | 0.64 | 613 | 0.36 | 0.84 | 0.71, 1.00 | 0.291 |
| Letra A. | 2012 | 620 | 426 | 0.69 | 194 | 0.31 | 564 | 348 | 0.62 | 216 | 0.38 | 0.73 | 0.58, 0.93 | 0.506 |
| Pooled |  |  | 0.68 (0.67, 0.70) | | 0.32 (0.30, 0.33) | |  | 0.65 (0.63, 0.66) | | 0.35 (0.34, 0.37) | | 0.84 (0.76, 0.93) | |  |
| **Mixed ethnicity** |  |  |  | |  | |  |  | |  | |  |  |  |
| Weatherley-White RC. | 2011 | 256 | 197 | 0.77 | 59 | 0.23 | 210 | 153 | 0.73 | 57 | 0.27 | 0.80 | 0.53, 1.23 | 0.896 |
| Bagordakis E. | 2013 | 466 | 332 | 0.71 | 134 | 0.29 | 616 | 421 | 0.68 | 195 | 0.32 | 0.87 | 0.67, 1.13 | 0.623 |

Supplementary Table 6

Allele Frequencies and Estimated Pooled Prevalence of Major and Minor Alleles for IRF-6 at SNPs 642961 by Ethnicity and Disease Groups

| Author | Year | CL/P | | | | | Control | | | | | A/G | | HWE |
| --- | --- | --- | --- | --- | --- | --- | --- | --- | --- | --- | --- | --- | --- | --- |
|  |  | No. of alleles | G allele | | A allele | | No. of alleles | G allele | | A allele | | OR | 95% CI | p value |
|  |  |  | Freq. | Prev. | Freq. | Prev. |  | Freq. | Prev. | Freq. | Prev. |  |  |  |
| **Asian** |  |  |  |  |  |  |  |  |  |  |  |  | |  |
| Pan Y. | 2010 | 254 | 188 | 0.74 | 66 | 0.26 | 230 | 195 | 0.85 | 35 | 0.15 | 1.96 | 1.24, 3.09 | 0.464 |
| Shi J. | 2011 | 346 | 262 | 0.76 | 84 | 0.24 | 312 | 265 | 0.85 | 47 | 0.15 | 1.81 | 1.22, 2.69 | 0.531 |
| Aldhorae KA. | 2014 | 484 | 392 | 0.81 | 92 | 0.19 | 840 | 723 | 0.86 | 117 | 0.14 | 1.45 | 1.07, 1.96 | 1.000 |
| Kerameddin S. | 2015 | 300 | 227 | 0.76 | 73 | 0.24 | 300 | 225 | 0.75 | 75 | 0.25 | 0.96 | 0.67, 1.40 | 1.000 |
| Pooled |  |  | 0.77 (0.74, 0.80) | | 0.23 (0.20, 0.26) | |  | 0.83 (0.79, 0.87) | | 0.17 (0.13, 0.22) | | 1.47 | 1.09, 1.98 |  |
| **Caucasian** |  |  |  |  |  |  |  |  |  |  |  |  | |  |
| Rahimov F. | 2008 | 736 | 515 | 0.70 | 221 | 0.30 | 2488 | 1941 | 0.78 | 547 | 0.22 | 1.52 | 1.27, 1.83 | 0.983 |
| Birnbaum S. | 2009 | 920 | 663 | 0.72 | 257 | 0.28 | 1902 | 1527 | 0.80 | 375 | 0.20 | 1.58 | 1.31, 1.90 | 0.064 |
| Mostowska A. | 2010 | 330 | 238 | 0.72 | 92 | 0.28 | 1130 | 882 | 0.78 | 248 | 0.22 | 1.38 | 1.04, 1.82 | 0.234 |
| Paranaiba LM. | 2010 | 354 | 301 | 0.85 | 53 | 0.15 | 252 | 216 | 0.86 | 36 | 0.14 | 1.06 | 0.67, 1.67 | 0.074 |
| Krasone K. | 2014 | 148 | 111 | 0.75 | 37 | 0.25 | 366 | 311 | 0.85 | 55 | 0.15 | 1.89 | 1.18, 3.02 | 0.085 |
| Pooled |  |  | 0.75 (0.69, 0.81) | | 0.25 (0.19, 0.31) | |  | 0.81 (0.78, 0.83) | | 0.19 (0.17, 0.22) | | 1.50 | 1.35, 1.68 |  |
| **Mixed ethnicity** |  |  |  |  |  |  |  |  |  |  |  |  | |  |
| Brito LA. | 2012 | 942 | 763 | 0.81 | 179 | 0.19 | 782 | 660 | 0.84 | 122 | 0.16 | 1.27 | 0.99, 1.63 | 0.013 |
| Borges AR. | 2015 | 586 | 501 | 0.85 | 85 | 0.15 | 704 | 627 | 0.89 | 77 | 0.11 | 1.38 | 0.99, 1.92 | 0.162 |

Supplementary Table 7

Allele Frequencies and Estimated Pooled Prevalence of Major and Minor Alleles for 8q24 at SNPs 987525 by Ethnicity and Disease Groups

| Author | Year | CL/P | | | | | Control | | | | | A/C | | HWE |
| --- | --- | --- | --- | --- | --- | --- | --- | --- | --- | --- | --- | --- | --- | --- |
|  |  | No. of | C allele | | A allele | | No. of | C allele | | A allele | | OR | 95% CI | P value |
|  |  | allele | Freq. | Prev. | Freq. | Prev. | allele | Freq. | Prev. | Freq. | Prev. |  |  |  |
| **Asian** |  |  |  |  |  |  |  |  |  |  |  |  | |  |
| Hikida M. | 2012 | 334 | 311 | 0.93 | 23 | 0.07 | 380 | 360 | 0.95 | 20 | 0.05 | 1.33 | 0.72, 2.47 | 1.000 |
| Xu MY. | 2012 | 432 | 402 | 0.93 | 30 | 0.07 | 400 | 374 | 0.94 | 26 | 0.07 | 1.07 | 0.62, 1.85 | 0.580 |
| Aldhorae KA. | 2014 | 484 | 271 | 0.56 | 213 | 0.44 | 842 | 564 | 0.67 | 278 | 0.33 | 1.60 | 1.27, 2.01 | 1.000 |
| Pooled |  |  | 0.81 (0.62, 1.00) | | 0.19 (0.004, 0.38) | |  | 0.85 (0.69, 1.01) | | 0.15 (0.00, 0.31) | | 1.48 | 1.21, 1.81 |  |
| **Caucasian** |  |  |  |  |  |  |  |  |  |  |  |  | |  |
| Birnbaum S. | 2009 | 924 | 572 | 0.62 | 352 | 0.38 | 1904 | 1520 | 0.80 | 384 | 0.20 | 2.44 | 2.05, 2.90 | 0.584 |
| Grant S. | 2009 | 222 | 141 | 0.64 | 81 | 0.36 | 11902 | 9343 | 0.78 | 2559 | 0.22 | 2.10 | 1.59, 2.77 | 1.000 |
| Nikopensius T. | 2009 | 434 | 310 | 0.71 | 124 | 0.29 | 2534 | 2131 | 0.84 | 403 | 0.16 | 2.12 | 1.67, 2.67 | 0.139 |
| Mostowska A. | 2010 | 330 | 224 | 0.68 | 106 | 0.32 | 1130 | 900 | 0.80 | 230 | 0.20 | 1.85 | 1.41, 2.43 | 0.715 |
| Pooled |  |  | 0.66 (0.61, 0.71) | | 0.34 (0.29, 0.39) | |  | 0.81 (0.78, 0.83) | | 0.20 (0.17, 0.22) | | 2.20 (1.96, 2.46) | |  |
| **Mixed ethnicity** |  |  |  |  |  |  |  |  |  |  |  |  | |  |
| Rojas-Martinez A. | 2010 | 298 | 249 | 0.84 | 49 | 0.16 | 606 | 531 | 0.88 | 75 | 0.12 | 1.39 | 0.94, 2.06 | 0.594 |
| Weatherley-WhiteRC. | 2011 | 258 | 147 | 0.57 | 111 | 0.43 | 210 | 126 | 0.60 | 84 | 0.40 | 1.13 | 0.78, 1.64 | 0.935 |
| Brito LA. | 2012 | 1334 | 858 | 0.64 | 476 | 0.36 | 1178 | 807 | 0.69 | 371 | 0.31 | 1.21 | 1.02, 1.43 | 0.148 |
| Velazquez JA. | 2012 | 264 | 220 | 0.83 | 44 | 0.17 | 740 | 625 | 0.84 | 115 | 0.16 | 1.09 | 0.74, 1.59 | 0.225 |
| Borges AR. | 2015 | 586 | 331 | 0.56 | 255 | 0.44 | 704 | 437 | 0.62 | 267 | 0.38 | 1.26 | 1.01, 1.58 | 0.224 |
| Pooled |  |  | 0.69 (0.58, 0.80) | | 0.31 (0.20, 0.42) | |  | 0.73 (0.62, 0.83) | | 0.27 (0.17, 0.38) | | 1.21 | 1.09, 1.36 |  |
